# The Live Attenuated Vaccine Strain “ARRIAH” Completely Protects Goats from a Virulent Lineage IV Field Strain of Peste Des Petits Ruminants Virus

**DOI:** 10.3390/vaccines12020110

**Published:** 2024-01-23

**Authors:** Olga Byadovskaya, Kseniya Shalina, Pavel Prutnikov, Irina Shumilova, Nikita Tenitilov, Alexei Konstantinov, Nataliya Moroz, Ilya Chvala, Alexander Sprygin

**Affiliations:** Federal Centre for Animal Health, Vladimir 600901, Russia

**Keywords:** peste des petits ruminants, goats, vaccines, Mongolia, ruminants, experiment, lineage

## Abstract

Peste des petits ruminants (PPR) is a transboundary viral disease that affects small ruminants, such as goats and sheep, in Africa, the Middle East, and Asia, causing substantial damage to livelihoods and disrupting livestock trade. Although Russia is PPR virus (PPRV)-free, controlling PPRV in neighboring countries is the top national priority. Recent PPR outbreaks in Mongolia and other countries in the Middle East caused by a lineage IV virus represent a risk of transboundary emergence in neighboring countries, including China, Kazakhstan, and Russia. In the present study, we assessed the potency and safety of the ARRIAH live attenuated PPRV vaccine (lineage II) in Zaannen and Nubian goat breeds by challenging them with a virulent lineage IV Mongolia/2021 isolate. For comparison, two commercial vaccines of Nigeria75/1 strain were used. The ARRIAH-vaccinated animals showed an increase in body temperature of 1–1.5 °C above the physiological norm, similar to the animals vaccinated with Nigeria75/1 vaccines. In all vaccinated groups, the average rectal temperature never exceeded 39.4–39.7 °C throughout the infection period, and no clinical signs of the disease were observed, demonstrating vaccine efficacy and safety in the current experimental setting. However, the control group (mock vaccinated) challenged with Mongolia/2021 PPRV exhibited moderate-to-severe clinical signs. Overall, the findings of the present study demonstrate that the ARRIAH vaccine strain has a promising protective phenotype compared with Nigeria75/1 vaccines, suggesting its potential as an effective alternative for curbing and controlling PPR in affected countries. Although the ARRIAH vaccine against PPR is not currently endorsed by the World Organization for Animal Health due to its incomplete safety and potency profile, this study is the first step to provide experimentally validated data on the ARRIAH vaccine.

## 1. Introduction

Peste des petits ruminants virus (PPRV) (sole member of species group *Morbillivirus caprinae*) causes a contagious and highly infectious respiratory disease in goats and sheep (https://www.woah.org/fileadmin/Home/eng/Animal_Health_in_the_World/docs/pdf/Disease_cards/PESTE_DES_PETITS_RUMINANTS.pdf (accessed on 17 September 2023)). This virus belongs to the family Paramyxoviridae and the genus Morbillivirus and has a single-stranded, negative-sense RNA [1]. Morbillivirus also includes measles virus, canine distemper virus, and rinderpest virus, a deadly but eradicated pathogen of cattle [2].

The genome of PPRV codes for two nonstructural proteins, C and V, and six structural proteins arranged in the order nucleoprotein (N), phosphoprotein (P), matrix protein (M), fusion protein (F), hemagglutinin (H), and viral RNA-dependent RNA polymerase (L) [3]. Among these, the N and F genes have been targeted as suitable loci for delineating the phylogeny of PPRV [4,5].

PPRV primarily affects sheep and goats and is manifested as an acute or subacute disease accompanied by fever, necrotic stomatitis, gastroenteritis, pneumonia, and death. Due to the associated dramatic economic losses, PPRV must be reported to the World Organization for Animal Health [6,7].

If neglected, PPR epidemics can destroy the entire sheep or goat population within a local settlement or a small region in a country [8,9,10], inflicting damage on the economy of households relying heavily on rearing small ruminants as an income source [11]. According to FAO estimates, the combined morbidity, mortality, production losses, and treatment expenses of PPR amounted to USD 2972.5 million/year during the period 2012–2017 across the affected countries of the South Asian Association for Regional Cooperation; in India alone, the amount was USD 2569.00 million/year [12].

At present, PPRV is widely spread in central, eastern, and western Africa; Asia; and the Middle East [1,13]. Four distinct lineages (I–IV) of PPRV have been identified based on its molecular epidemiology [14]. In terms of geography, lineages I and II are restricted to western and central Africa, lineage III is found in eastern Africa and the Arabian Peninsula, and lineage IV is found in Asia (China and Mongolia); South Asia; the Middle East; and northern, western, central, and eastern regions of Africa [15,16,17]. Lineage IV has now assumed the widest endemic profile and appears to be evolving and spreading rapidly [1,18].

The most efficient way to combat PPRV is mass vaccination. Several live attenuated vaccines have been developed and recommended for PPRV control worldwide [19]. Although the most extensively used and WOAH-endorsed vaccine is the Nigeria75/1 strain belonging to lineage II cluster [20], some countries opt for local strains to prepare a vaccine and administer it locally, provided their potency and efficacy are demonstrated [21]. In India, vaccine strains encompassing Sungri 96, Arasur 87, and Coimbatore 97, which all belong to lineage IV, have been developed, with Sungri 96 being nationally used in mass PPRV vaccination campaigns [21,22]. In Bangladesh, another live attenuated vaccine (Titu strain) was developed by the Bangladesh Livestock Research Institute and has been successfully used to control PPRV for the last 20 years [23,24]. However, these vaccines lack WOAH endorsement due to insufficient data available for their international usage.

The Federal Center for Animal Health in Vladimir, Russia, manufactures a PPRV vaccine known as the “ARRIAH strain,” which belongs to lineage II and currently lacks WOAH endorsement. In contrast to other countries where PPR vaccines are locally produced for domestic use, the ARRIAH vaccine was developed to control PPR in neighboring countries with a history of PPR outbreaks that could potentially spill over and jeopardize their official status. Since no PPR cases have been reported to date in Russia, it was certified PPRV-free by WOAH in 2019. However, controlling PPRV in neighboring countries has now become the top national priority. For this reason, internationally recognized and officially endorsed vaccines with proven potency and efficacy against PPRV are needed, as is the case for the already established vaccine Nigeria 75/1, to be marketed internationally to readily curb outbreaks or ascertain freedom with vaccination in different parts of the world. As PPRV is a transboundary threat, the task of eradication via vaccination should be accomplished effectively and promptly due to the current situation. A previous analysis of the current epizootic situation of PPR in countries adjacent to Russia indicated a high degree of disease threat in the Siberian, Ural, and Far Eastern regions and a low degree of threat in the North Caucasian and Southern federal districts with pasture management of livestock [25].

As a part of our preliminary efforts to promote the ARRIAH strain for official endorsement, this study focuses on assessing the efficacy and safety of the live attenuated strain of PPRV “ARRIAH” against the virulent Mongolian PPRV isolate (lineage IV), which caused a PPR outbreak in Mongolia in 2021 [17].

## 2. Materials and Methods

### 2.1. Vaccine Strains

In this study, we assessed the efficacy of the ARRIAH vaccine strain (designated as V1, FGBI ARRIAH, Vladimir, Russia), which was previously identified to be related to 45G37-k (Federal Research Center for Virology and Microbiology, Pokrov, Russia) and to share the same origin (lineage II) [26]. The efficacy of the ARRIAH vaccine strain was evaluated against two commercially available batches, each from a different manufacturer, containing the Nigeria75/1 vaccine strain. Due to ethical concerns, the manufacturers were anonymized and designated as V2 and V3. The V1 vaccine had a dose of 2.5 log10 TCID50/does per 1 mL, as per the manufacturer’s recommendation. The recommended inoculum doses for V2 and V3 were 1 mL and 1 mL, respectively, both with a dose of 2.5 log10 TCID50/does, as per the manufacturers’ instructions. The animals were vaccinated subcutaneously in a wool-less area of the hind leg, following the manufacturers’ recommended route of administration.

### 2.2. Challenge Stain

The Mongolia/2021 isolate belonging to lineage IV was used for the challenge experiment. The strain was isolated from the 2021 PPR outbreaks in Mongolia and submitted to FGBI ARRIAH for confirmation as a member of the WOAH Reference Laboratory Network [17]. The field strain was passaged three times on continuous goat gonad cells to ensure that the inoculum was free of secondary microflora. The challenge inoculum was 3.5 log10 TCID50/does per 1 mL. The experimental goats were subcutaneously challenged with this strain on day 21 post vaccination (pv).

### 2.3. Experimental Design

Animal experiments were conducted in high-security containment animal units at the Federal Center for Animal Health (FGBI ARRIAH, Vladimir, Russian Federation). For these experiments, 20 heads of 6–9-month-old goats of different sexes, including 12 heads of the Zaannen breed and 8 heads of the Nubian breed, were used. Upon arrival, all animals were inspected to be healthy and disease free.

The rectal temperature of all animals was within the normal range (38.4–39.0 °C). The housing facility complied with biosafety 3 conditions and was insect-proof.

The animals were divided into four groups: one control group for the challenge and three experimental groups (V1, V2, and V3 groups) for vaccine administration, with five animals (two Nubian and three Zaannen breed individuals) in each group.

### 2.4. Ethics Statement

The care and handling of animals were performed according to the Guidelines of the European Union Council (2010/63/EU). This study was submitted to, reviewed, and endorsed by the Committee on ethics of the Federal Center for Animal Health, Russia (Permit Number: No. 6/9-05042023). The animals were housed in a BSL3 animal facility. To relieve the pain and suffering of animals, they were subjected to euthanasia by captive-bold penetration, after which the muscle relaxant Adilin Super was intravenously administered (Federal Center for Toxicological, Radiation, and Biological Safety, Kazan, Russia). Adilin Super was injected at the dose recommended by the manufacturer, which was equal to 5 mg/kg (according to the instructions for use), in compliance with the Russian Federal Service for Veterinary and Phytosanitary Surveillance decree in 2008.

### 2.5. Clinical Monitoring

The vaccinated and challenged animals were observed twice daily throughout the experimental period. The monitored clinical signs included changes in behavior, appetite, nasal and ocular discharge, lymph node enlargement, diarrhea, and fever, based on the published clinical score system [27].

Animals from both the experimental (vaccinated) and control (nonvaccinated) groups underwent regular clinical examinations and temperature measurements for 21 days. On day 21 after vaccination, both the experimental and control groups were challenged. Over 14 days following the challenge, all animals were closely monitored. Clinical signs were recorded, temperatures were measured (Table 1), and samples were collected regularly for real-time reverse transcriptase PCR (RT-PCR).

The clinical conditions of the animals were assessed by considering the various indicators (body temperature, vaccine injection site, overall well-being, changes in the mucus epithelium of the mouth, changes in the mucus epithelium of the nose, changes in the conjunctiva, enlargement of lymph nodes, gross pathology in the respiratory tract, and gross pathology in the gastrointestinal tract), with each indicator assigned a specific score (Table 1). These scores were recorded upon the initial identification of each indicator. By summing up the scores for each group, we indirectly evaluated the extent of post vaccination reactions within the population when using a specific vaccine preparation.

All animals were monitored for 14 days after infection. The severity of the disease course was also assessed in points.

### 2.6. Assays

To monitor viral RNA release and viremia progression, blood and eye swabs of the vaccinated animals were tested before vaccination; on day 3, 5, 7, 14, and 21 pv; and on day 0, 3, 5, 7, 9, 11, and 14 post challenge, followed by collection in 100 µL of lysis buffer (Kit “Lira+” for RNA and DNA isolation (LRP-100-2), Russia). All blood samples were collected from the jugular vein of the goats using an adapter system.

The collected samples were stored at −70 °C until further processing. Total RNA was extracted from the blood and ocular samples using the viral extraction kit Lira+ (Kit “Lira+” for RNA and DNA isolation (LRP-100-2), Russia) following the manufacturer’s instructions. The extracted RNA was eluted in a final volume of 25 µL.

RT-PCR was conducted using a commercial real-time RT-PCR kit (FGBI ARRIAH), recognized as suitable for this purpose to detect all linages by the reference WOAH laboratory (CIRAD, France) in an independent trial. In brief, 8 µL of the template was added to the reaction mixture, which contained 7 µL of RNase-free water, 5 µL of a 2× RT-PCR buffer, 0.5 µL of a 25× RT-PCR enzyme mix, and 1.0 µL of a specific primer probe mix.

All Ct values of up to 37 were considered positive. The PCR machine used was Rotor Gene Qplex (Qiagen, Germany), which employs an FAM channel. While all samples were tested twice, any discrepancies between the two results triggered confirmation through repeat testing.

For serum collection, the tubes were centrifuged at 3000× *g* for 10 min and were stored at −80 °C until analysis. Serum samples were evaluated at 0, 7, 14, and 21 dpv using ID Screen PPR competition enzyme-linked immunosorbent assay (ELISA IDvet, Montpellier, France) following the manufacturer’s instructions. The ELISA results were considered as follows: SP ≤ 50%, positive; SP > 60, negative. Following the challenge, seroconversion was evaluated at 7 and 14 days post infection (pi).

## 3. Results

Symptoms

After 21 days of observation following vaccination, the average group scores in the experimental groups remained consistent, ranging from 0.9 to 1.1 points.

After the challenge with the virulent isolate “Mongolia/2021”, the following clinical signs were observed in animals in the control group:

– An increase in rectal temperature to 40–41 °C was observed at 2–4 dpi;
– Apathy and refusal of feed was observed on 3–6 dpi; moreover, the animals lied down a lot and rose reluctantly;
– An increase in the size of lymph nodes was recorded at 5–7 dpi, and serous discharge from the nostrils appeared in some animals;
– At 6–9 dpi, signs of damage to the respiratory system began to appear: serous discharge from the nostrils, hyperemia of the nasal mucosa, wheezing when breathing, coughing, and shortness of breath after running; animals no. three and four lied down and had a cough, shallow breathing, and high body temperature;
– At days 10–12, animals no. three and four were euthanized due to their severe clinical condition (bilateral pneumonia). The remaining animals in the control group showed pronounced clinical signs of respiratory pathology. In addition, diarrhea was recorded in one animal.

The animals of the experimental vaccine groups V1–V3 did not show any clinical signs throughout the observation period. In single animals, a short-term increase in rectal temperature at 2–3 dpi was observed after virus inoculation, which lasted less than a day.

**Figure 1:**
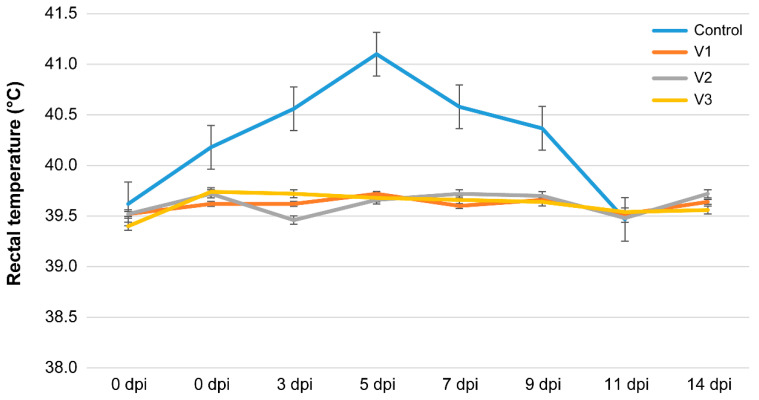
Kinetics of the average rectal temperature values for each group during the experimental period. The error bars of each time point designate one standard deviation.

During the adaptation period (the first week after arrival), the rectal temperature of the animals was within the normal range (38.5–39.5 °C) (Figure 1).

Animals in the experimental vaccine groups (V1–V3) showed a slight increase in body temperature, averaging 0.5–1.0° above the physiological norm on the second or fourth day. No changes were observed at the vaccine injection site. The physiological parameters of the control group animals remained normal (Figure 1).

After challenging the control group, their average rectal temperature increased from 39.2 °C to 41.0 °C, whereas the peak temperature was >41 °C at 5 dpi and remained at 40.4 °C up to 11 dpi.

In the vaccinated groups, the average rectal temperature never exceeded 39.4–39.7 °C throughout the infection period and no clinical signs of the disease were observed following the challenge.

### 3.1. Detection of PPRV RNA in Blood Samples and Nasal Swabs

**Figure 2:**
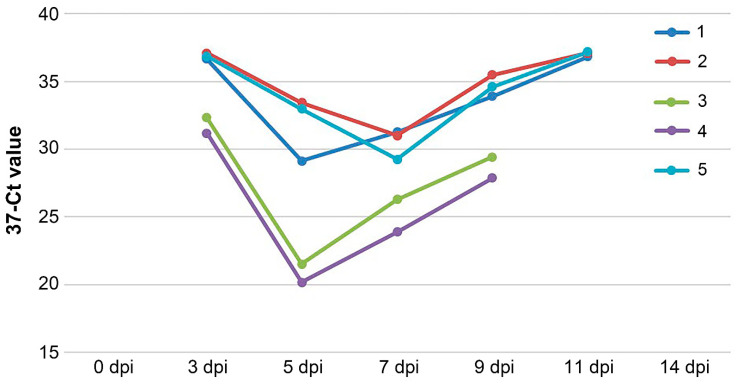
Dynamics of the detection of PPRV RNA in the nasal swabs of the control group (unvaccinated and challenged) via RT-PCR during the experiment.

**Figure 3:**
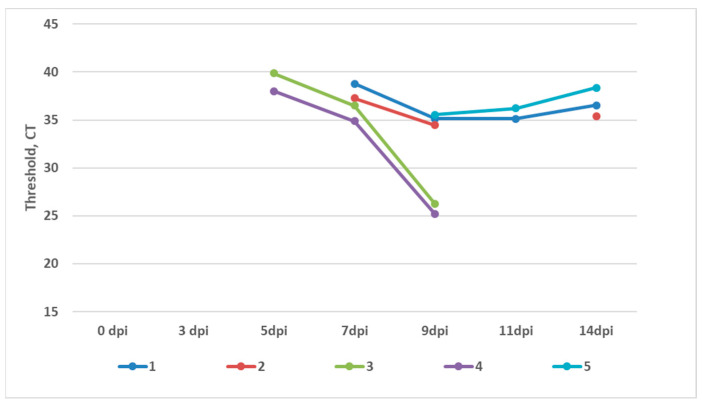
Dynamics of the detection of PPRV RNA in the blood of the control group (unvaccinated and challenged) via RT-PCR during the experiment.

Whole blood samples (with EDTA) and smears from the nasal mucosa were collected from the animals after vaccination and infection. RT-PCR was performed to detect PPRV RNA. The absence of viral RNA in the vaccinated animal groups (V1–V3) post vaccination and infection suggests that the ARRIAH vaccine is as safe as the commercial Nigeria75/1 vaccines, providing sterile immunity. In contrast, viral RNA was detected in all of the sample types from the unvaccinated control animals (K) at 3 dpi in mucosal smears and at 7 dpi in whole blood samples (Figure 2 and Figure 3). Virus shedding lasted from 3 dpi till 11 dpi, while viremia lasted from 7 dpi till 14 dpi. This demonstrates the virulence of the Mongolia/2021 strain, validating its suitability for assessing the tested vaccine.

### 3.2. Detection of Antibodies against PPRV

**Figure 4:**
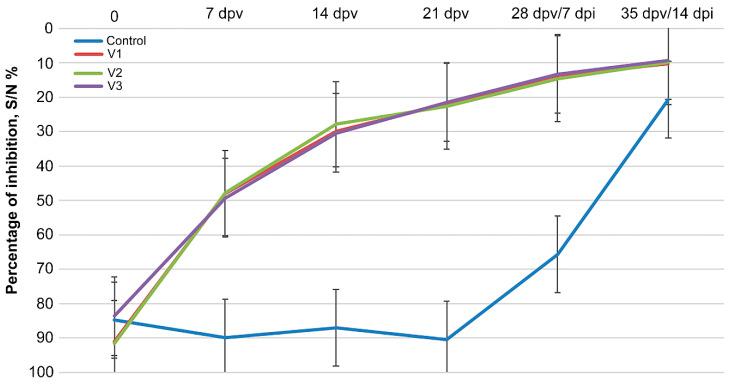
Detection of antibodies against the N-protein of PPRV in the sera of the experimental and control groups after vaccination and infection using ELISA. The results are presented as the average values of the percentage of inhibition (S/N%) for the group (n = 5), with error bars representing one standard deviation.

Before vaccination and at 7, 14, and 21 dpv, antibodies against the viral nucleocapsid protein (N) were determined in animals of the experimental groups (V1–V3) using a commercially available ELISA kit (IDVET). On the day of vaccination (day 0), all animals were seronegative for anti-N antibodies. In general, all vaccines induced antibodies against the N-protein (Figure 4). A week after vaccination, the animals of all three groups had equivalent levels of detectable specific antibodies, which increased 2 weeks after vaccination and then persisted for the rest of the study period (Figure 4). On the day of infection (3 weeks after vaccination), the level of antibodies in the range of 21.4–22.6% of inhibition was observed in all experimental group animals (V1–V3).

## 4. Discussion

The intensification of cross-border trade relations and regional wars contributes to the spread of transboundary diseases [28]. PPRV is an economically important pathogen whose distribution threatens the economy of households relying largely on sheep or goat rearing as an income source [29,30]. To achieve the goal of complete PPR eradication by 2030, the PPR Global Control and Eradication Strategy was established and is being implemented [31]. A crucial component of this strategy involves successful vaccination campaigns worldwide, relying heavily on current knowledge about the performance of available vaccines.

Similar to smallpox and rinderpest, PPR eradication is primarily targeted through vaccination. Nigeria 75/1 and Sungri 96 are the most widely used vaccines with sufficient data on their efficacy, safety, and potency. Moreover, Nigeria 75/1 is the only vaccine with proven efficacy and has been used in several countries. Both vaccines have been shown to protect against all genetically defined lineages [32]. Other PPRV live attenuated vaccine strains have also been developed and successfully used worldwide [23].

Analysis of the epizootic situation of PPR and recent trends of disease spread in countries adjacent to the Russian Federation, performed using cartographic materials and official data sources, indicates a high degree of threat of PPRV emergence in the territory of the Russian Federation. In particular, the epizootic situation in Mongolia, China, Georgia, Turkey, and Iran should be focused on [25]. Consequently, the risk of PPRV incursion or spillover from countries in which PPRV is prevalent remains to be high [25]. Based on the results of the risk assessment, possible directions of introduction of PPRV into the Russian Federation, which is free of PPRV, accounting for the geographical location of affected states, are as follows: Turkey, Iran, and Georgia, the Caucasian direction; the Republic of Kazakhstan and Western China, the Siberian direction of drift; and Mongolia and Eastern China, the Far Eastern direction of drift [25]. This risk warrants the availability of highly sensitive diagnostic tools and stockpiles of emergency vaccines.

The ARRIAH strain produced by FGBI ARRIAH belongs to lineage II, as the Nigeria 75-1 vaccine strain does, but is not directly related to the wild Nigeria strain at the origin of this vaccine [26]. Although it still lacks WOAH endorsement, this vaccine has been used in some central Asian countries to control PPRV spread [33,34,35].

In this study, we successfully assessed the ARRIAH strain against a virulent PPR strain detected in Mongolian sheep in 2021 in a controlled setting in goats [17]. The experiment also included two Nigeria75/1 vaccines from different manufacturers. We chose Zaannen and Nubian goat breeds due to their prevalence in Europe, north and central Africa, and Asia. Furthermore, their susceptibility to PPR infection makes them essential subjects for experimental vaccine studies.

The Mongolia/2021 strain proved virulent under experimental conditions and caused detectable viremia and virus shedding in control animals, unvaccinated animals, followed by the manifestation of clinical disease (Table 2). This corroborates a study reporting that PPRV RNA can be detected as early as 3 dpi. However, in contrary to this same study, viral RNA was not detected in swabs at 14dpi, while a low level of RNA was still detected in the blood at 14dpi. Additional sampling points and tests using other RT-PCR methods would be needed to explore further the observed differences [36,37,38].

Although our study is not the first to evaluate the efficacy of the ARRIAH vaccine strain in a controlled setting [33,34], it revealed that the ARRIAH strain and Nigeria75/1 vaccines were equally efficient against the virulent Mongolian strain in a controlled experiment. Comparative analysis of the ARRIAH vaccine strain and two Nigeria 75/1 vaccines from different manufacturers against a virulent lineage IV strain demonstrated 100% protection in all vaccinated goats, regardless of the vaccine type or breed. In addition, no RNA shedding was detected in the V1, V2, or V3 goats (Figure 3 and Figure 4).

The ARRIAH vaccine strain differs from Nigeria75/1. A recent study revealed that a PPRV vaccine strain, 45G37-k (Pokrov, Russia), differed from Nigeria75/1 by 248 nucleotides; however, the ARRIAH strain was related to 45G37-k, indicating the shared origin. These lineage II variants likely originated from a distinct lineage II field isolate that circulated alongside the predecessor of Nigeria 75/1 in the past [26]. Although Nigeria 75/1 was attenuated on 75 passages in Vero cells [20], the attenuating mutations accrued during this passage history differ from those of the ARRIAH and 45G37-k strains (35 passages in lamb kidney cells for ARRIAH and serial passages in lamb kidneys, lamb testes, and Saiga kidney cells for 45G37-k) [26]. Unfortunately, the complete passage history or the exact date and place of collection of the field isolate is not available.

Several live attenuated vaccines were previously developed using a haphazard method for viral attenuation [39,40,41]. The term “haphazard” was used because the growth conditions for the wild-type counterpart were potentially different, e.g., Vero cells (green monkey) for PPRV and chicken embryos for Neethling LSDV [20,42]. This trial-and-error approach prompted different attenuations of the same virus using the same protocol, resulting in different evolutionary trajectories. In our case, this is the ARRIAH strain and Nigeria 75/1, although both of which likely had different ancestors in the past [26]. Despite exhibiting different genome compositions, both strains demonstrated equal efficacy against PPR in this experiment; this finding was consistent with that of previous studies (Figure 4) [32,43]. Notably, this controlled experiment is the first step toward formal international recognition of the ARRIAH strain for use against PPR. This ARRIAH vaccine has been used in Mongolia and Tajikistan to fight PPR outbreaks and control the situation along the borders with endemic countries.

One limitation of this study is that it only provides information on efficacy against the lineage IV PPR virus that currently poses the chief risk of introduction into the Russian Federation. Protection against viruses of other lineages has not yet been confirmed but will be required for its overall recognition for international usage. In conclusion, we evaluated the virulent lineage IV PPRV strain Mongolia/2021 against the ARRIAH vaccine strain and two other Nigeria 75/1 vaccines. The obtained findings revealed that the ARRIAH vaccine strain can offer a similar level of protection as the widely used Nigeria 75/1 vaccines, despite having different attenuating mutations. More experimental studies are needed to established the utility of the ARRIAH vaccine in a wider animal population under experimental and field settings. This research paves the way for further investigations to establish the safety and efficacy of the ARRIAH vaccine at different doses and reversal potential, aiming for recognition in PPR eradication campaigns under the WOAH and FAO's PPR Global Control Eradication Strategy [31]. For vaccine development and PPR eradication, conducting monitoring studies in the border regions of high-risk infection zones (e.g., the Republics of Tyva, Altai, Buryatia, Zabaikalsky Krai, Amur Region, EAO, and Primorsky Krai) is necessary to determine the presence of PPR among susceptible domestic livestock [25].

## 5. Conclusions

Overall, in this study, we evaluated the ARRIAH vaccine in Zaannen and Nubian goat breeds challenged with a virulent currently circulating lineage IV Mongolia/2021 isolate. When compared with Nigeria75-1 vaccines, the ARRIAH vaccine performed well by conferring complete protection without vaccine-related viremia and side effects, while the control animals succumbed to infection. The obtained findings are the first on the way toward international recognition of the ARRIAH strain vaccine.

## Figures and Tables

**Table 1:** Assessment of clinical signs upon occurrence.

Symptom		Score
Body temperature	>40 °C (every 24 h)	0.5
	>41 °C (every 24 h)	1
Vaccine injection site	Swelling over 3 cm in diameter; redness and pain for 12 h	1
	Pathological changes in the skin or underneath	2
Overall well-being	Apathy, weakness, and loss of appetite (every 24 h)	0.5
	Reluctance to get up (every 24 h)	2
	Death	6
Changes in the mucus epithelium of the mouth	Hyperemia	1
	Erosive lesions; hypersalivation	2
	Necrosis	3
Changes in the mucus epithelium of the nose	Hyperemia; nasal discharge	1
	Erosive lesions	2
Changes in the conjunctiva (conjunctivitis and serous discharge)		2
Enlargement of lymph nodes		1
Gross pathology in the respiratory tract		3
Gross pathology in the gastrointestinal tract		3

**Table 2:** Scores following vaccination and challenge.

Group	Control	V1	V2	V3
Average score after vaccination	0	1.0	0.9	1.1
Average score after challenge	15.2	0.1	0.2	0.1

## Data Availability

Data generated or analyzed during this study are included in this published article. Correspondence and requests for materials should be addressed to A.S.
